# A qualitative study on primary health care professionals’ perceptions of mental health, suicidal problems and help-seeking among young people in Nicaragua

**DOI:** 10.1186/1471-2296-15-129

**Published:** 2014-07-02

**Authors:** Claudia Obando Medina, Gunnar Kullgren, Kjerstin Dahlblom

**Affiliations:** 1 Centre for Demography and Health Research, Nicaraguan National Autonomous University, León, Nicaragua; 2 Department of Clinical Sciences, Division of Psychiatry, Umeå University, Umeå, Sweden; 3 Department of Public Health and Clinical Medicine, Division of Epidemiology and Global Health, Umeå University, Umeå, Sweden

**Keywords:** Young people, Help seeking, Primary health care, Nicaragua, Qualitative method

## Abstract

**Background:**

Mental health problems among young peoples are a growing public health issue around the world. In low- income countries health systems are characterized by lack of facilities, human resources and primary health care is rarely an integrated part of overall health care services. This study aims at exploring how primary health care professionals in Nicaragua perceive young people’s mental health problems, suicidal problems and help–seeking behaviour.

**Methods:**

Twelve in-depth interviews were conducted with nurses and doctors working in primary health care services in León, Nicaragua. A qualitative research design was applied. Data was analysed using thematic analysis approach.

**Results:**

This study revealed that doctors and nurses were reluctant to deal with young people presenting with suicidal problems at the primary health care. This was more likely to stem from feelings of incompetence rather than from negative attitudes. Other barriers in providing appropriate care to young people with mental health problems were identified such as lack of time, lack of privacy, lack of human resources, lack of trained professionals and difficulties in communicating with young people. The primary health care (PHC) professionals suggested different solutions to improve care for young people with suicidal problems.

**Conclusion:**

PHC doctors and nurses in Nicaragua felt that providing skilled mental health services to young people was a priority for them but they also identified a number of barriers to be able to do so. They discussed ways to improve young people’s willingness to share sensitive issues with them and suggested ways to make PHC more appreciated by young people.

## Background

Health is defined in terms of both mental and physical well-being, implying that mental health is an important part of health care. Mental disorders represent more than 10% of the overall disease burden worldwide, affecting people of all ages, cultures and at all socioeconomic levels [1]. Still, investment in mental health services is neglected, particularly in low- and middle-income countries, where 85% of the world’s population live [2]. There seems to be a long way to go until health systems can provide an “equitable, inclusive and fair” model integrating mental health. A primary health care (PHC) system well integrated in the community and accessible to all individuals has a key role to play in promoting mental health and the treatment of common mental disorders [3].

Suicidal problems represent one area where PHC interventions can make a difference. Suicide victims and attempters are likely to have attended health care services presenting with other health problems prior to their suicidal act. A review of 40 studies has shown that an average of 45% of suicide victims were in contact with primary care providers within one month before committing suicide. Counselling skills can help health care professionals identify those at risk [4]. For those high-risk individuals who present with overt suicidal behaviour, proper management is crucial.

There are many barriers for PHC in managing these challenges. A lack of resources and money is not always the main obstacles [2]. A more significant barrier might be a lack of appropriate structures for the management of suicidal patients, or the attitudes of staff towards such patients. The attitudes of patients themselves could be another barrier, since people exhibiting suicidal behaviours often try to solve their problems on their own, before seeking professional help [5].

A systematic review of 31 studies in Western countries on attitudes towards clinical services among people with self-harm behaviour showed that patients had a negative perception of how they were treated. Despite differences between countries and health care systems, there was a common perception that there was a lack of patient involvement in management decisions, inappropriate staff behaviour and a lack of knowledge among staff [6]. Several studies have explored factors associated with the negative attitudes of staff and their unwillingness to engage with patients showing suicidal behaviour. Lack of adequate training is reported to be associated with negative attitudes [7-9]. Age and length of clinical experience play a role. For example, an Irish study among nurses from an emergency department identified a trend whereby initially positive attitudes reached a peak after which they declined and became less positive [10]. Similar findings have been reported by Friedman and co-workers [7]. Inter-professional problems, such as poor communication between nurses and doctors, can be another obstacle [11]. Doctors in rural hospitals felt that relations with other staff members functioned as a barrier to the appropriate management of suicidal patients [12]. Misconceptions might also be a barrier; a study in primary health care found that only one out of 52 general practitioners believed that suicide was preventable [13].

### PHC services and mental health in Nicaragua

In Nicaragua mental health has not been prioritized by the health system. There are few human and economic resources available, for example, there are only 0.64 psychiatrists per 100,000 inhabitants [2] while the minimum ratio is 1:10,000 psychiatrists as suggested by the American Psychiatric Association [14]. In León, a city with 200.000 inhabitants, there are only two psychologists shared by three primary health care centres, and only one psychiatrist who works in the general hospital. In the PHC centres, general physicians provide for mental health care but overall PHC professionals have little or no training in psychiatry [15]. There is one psychiatric outpatient unit, Centro de Atención Psicosocial (CAPS). Patients are referred to CAPS through the PHC or the hospital.

There has been a few training workshops for health and community services personnel based on a manual developed by Pan American Health Organization (PAHO) and the Minister of Health in 2003 which offers guidelines on how patients at risk of suicide should be approached. The manual deals with staff attitudes at the different levels of attention, precautions against suicide, diagnosis and follow-up for the care of patients. It also addresses community participation and coordination with other institutions [16].

The system of community care for the mentally ill includes preventive/promotional interventions, home interventions, family interventions, residential facilities, and vocational training. However, these are only available for less than 25% of the population [2]. Community care is available at PHC level with monthly home visits, where nurses and sometimes a doctor visit families in their homes, asking and informing about health issues.

This study aims at exploring how primary health care professionals perceive young people’s help–seeking behaviour in Nicaragua, a low-income country. It aims specifically at understanding PHC professionals’ perceptions of mental health and suicidal problems among young people.

## Methods

### Setting

The study was carried out at three primary health care centres (PHCC) in León, Nicaragua. León is located in the North Western Pacific region of Nicaragua and is the second most important city in the country. It is a university town with a rich historical past. On average, a PHCC serves 36,000 people with a wide variation between regions. On average, there are 4.5 doctors, 3.4 registered nurses and 7.1 auxiliary nurses per PHCC [17].

### Study design

An emergent qualitative design was applied; data were gathered through observations at primary health care centre’s and from individual interviews with primary health care staff in urban León. The unstructured observations were conducted in order to become familiar with the setting and to gain a deeper understanding of the context.

Semi-structured interviews were conducted with nurses and general practitioners in the three main public primary health care centres in León municipality. Five nurses and seven general practitioners with a mean average of twelve years working in the health system participated.

We used stratified purposive sampling to select the informants [18]. Criteria for inclusion in the study were: being a nurse or doctor, and being in contact with adolescents and young people in their everyday work. We contacted the director of each primary health care centre and in total 22 informants were identified and invited to participate. Those that declined did so because of time constraints.

A psychologist, also the first author (CO) of this paper, contacted the selected persons at their workplace in the primary health care centre to have an initial meeting and set up an appointment for an interview. All interviews were conducted by the principal researcher (CO) and took place in a room free from disturbances in the PHC where the informant worked.

Twelve consecutive in-depth interviews were conducted. The emergent design is an interactive process of data collection and analysis, allowing for modifying and exploring emerging topics with the next informant. We limited the number of interviews to twelve to be able to analyze the rich data in-depth. The analysis started after the first interview, and the first author transcribed all interviews after each session so that the text could be read and scrutinized continuously.

### Interview guide

An interview guide with open-ended questions was used to explore the perceptions and attitudes towards help-seeking and suicidal behaviour among young people. The questions focused on their perceptions and views towards young patients with suicidal ideation, how these patients were managed in their setting and their familiarity with the national guidelines on the management of patients with suicidal ideation. An introductory vignette was used to stimulate response. After reading the vignette the informants were asked for example: “What would you do in this situation?” “How would you feel as health care professional?”

### Description of vignette

The vignette provided information about an 18-year-old boy with alcohol problems. It described that his father was violent and abused both him and his mother. Because of the violence the parents had separated. He had few friends and was no longer in school. He had exhibited somatic symptoms in previous contacts with medical staff, who had just given him prescriptions for headache, stomach and back pain. He had made previous suicide attempts and one day he had shown up in the PHC centre, where he suddenly started to cry and express that he felt alone and tired of life.

### Analysis

A thematic analysis approach was used; it is a flexible qualitative method that identifies, interprets, analyses and reports themes derived from data [19]. Through a systematic process the data is coded and organized, involving the identification of themes through careful reading and re-reading of the transcribed interviews. The interpretation process is interactive and reflexive, where the researchers identify themes which are recurrent and “adequately reflect their textual data” [20]. Janice Morse describes a theme as a meaningful “essence” that runs through the data as a basic topic that the narrative is about [21]. In this study the research team, consisting of a psychologist, a social scientist and a psychiatrist, discussed and negotiated the themes after having analysed the data individually. Colleagues reviewed findings and interpretations in order to increase trustworthiness.

An example of the data analysis is presented in Table 1. The direction of the process is from quotes to themes via codes. Quotes generate codes who in turn identifies themes.

**Table 1:** Example of the process of analysis

**Examples of quotes**	**Codes**	**Theme**
*The other thing is the fact that I can’t address [the problems] in five, ten, fifteen minutes, because first of all you have to create a climate of confidence. Because if you’ve got a patient here, you’ve got another one waiting back there and sometimes you make the mistake of rushing.”*	● Lack of time	Barriers to help young people
	● Lack of training/knowledge	
	● Lack of skills to detect problems ● Building trust takes time	
	● Stressing to keep schedule	
*“The number of patients per doctor is quite large. I’m sure that if we had a little more time, problems could be addressed… I believe that we need more staff trained in mental health… sometimes the authorities believe that health problems are only physical, and mental health is put a little behind and it shouldn’t be like that because in this country… with so many problems that we have….for example just in two of the big PHCs there is only one psychologist and no psychiatrist.* *I’m not saying that doctors can’t do it - on the contrary - I think that doctors should always be educated and trained in mental health problems.”*	● Lack of time	Barriers to help young people
	● More human resources	
	● Mental health is not a priority	
	● Not given the real importance	
	● Lack of training	
	● Frustration	
	● More training in MH	
	● Looking to improve their skills	
	● Open attitude to learn	
*“…sometimes it becomes very difficult to meet here. We do not have adequate space, we don’t have the privacy that patients need to highlight the problems they have, and I think that is why people do not give us all the information.*	● Lack of privacy	Barriers to help young people
	● Lack of time	
	● Lack of skills	
	● “Hot potato”	
	● Caring for the patients	
	● Inadequate conditions	
	● Referring to others	
*But even with all those limitations, I’m trying to do my best, so I listen to their problems and then refer them to the psychologist because it requires more time… to try to solve the family problems they have. We can’t do it here, so it’s better to refer to the psychologist and the psychologist can give them more time and they can solve it.”*		

### Ethical considerations

The research study was approved by the Ethics Research Committee of UNAN-León University, Nicaragua and Umeå University (07–046 M), Sweden. All participants gave their written informed consent to be interviewed. The interview began with a presentation of the interviewer and the aim of the study. Each informant was encouraged to talk freely and in detail about the issues regarding their experiences with adolescents and young people. Interviews lasted about one hour and were recorded and then transcribed.

## Results

Six key themes were extracted from the interview data; “Building trust takes time”, “Preservers of life”, “Avoiding ‘the hot potato’”, “Dealing with frustration and powerlessness”, “Barriers to helping young people”, and “Identified needs for suicide prevention”.

### “Building trust takes time”

In general, doctors and nurses perceived that young people mainly come to primary health care seeking help with issues linked to reproductive health, such as problems related to contraceptive use or pregnancy. All informants mentioned trust as a key aspect in their everyday work with their patients, and emphasised that trust is a key issue for all actors involved,: the community, the primary health care centre and young people attending the centre Several informants commented that age difference between themselves and their young patients had very little to do with building a positive relationship with young patients.

“Young people don’t come often to the centre for mental health issues or sensitive issues, but they come for condoms, family planning protection and pregnancy care. In that sense they trust us and after a while they can be more open with us.” (Female doctor )

### “Preservers of life”

The informants emphasised that they were trained to save lives and to act as preservers of life. They expressed a strong belief in the value of life. This influenced their way of looking at young people with suicidal problems. However, they were aware of having insufficient knowledge of psychology, and had difficulties understanding young people showing suicidal expressions.

“Let me tell you something that maybe sounds bad, but it’s the reality and many other colleagues have the same opinion as well: when I started to study medicine I had the idea that we are here to help people, help them to live… right? …And that is what we deal with every day - saving pregnant women or elderly people’s lives, …” (Male doctor)

Negative attitudes towards suicide patients were justified by saying, for example:

“In practice we get more work and we need to prioritize patients; I mean I have to choose between patients with a disease and someone who wants to die.” (Male doctor)

### “Avoiding the ‘hot potato’”

In exploring perceptions of mental health and help-seeking at primary health care centres, it emerged that nurses and doctors felt like they were only one of the links in the chain in the treatment process. Nurses reflected that they did not have enough training to take care of mental health problems. Usually a nurse would refer the patient to a doctor (general physician) who, in turn, would refer the patient to a psychologist or psychiatrist with the same argument.

“I have no training in taking care of mental health problems” “I do all I can do, if I cannot help I will refer the patient.” (Female nurse)

We use the metaphor of “the hot potato” (papa caliente) to describe this pattern of handing over responsibility of the patient to another professional. No one wants to deal with the problematic patient and tries to avoid difficult situations.

“…I do not want to deal with these kinds of patients, I refer them to someone else.” “…I don’t have the time to listen and solve their problems.” (Male doctor)

Not all informants shared this view; some of them were more flexible with time during their consultations and recognized that patients with somatic symptoms must have a closer examination that also looks at social and family problems. They wanted to help these patients but felt they did not have enough training and needed to develop skills in how to manage patients at risk for suicide.

“You need skills to work with these patients - not everyone can work with these kinds of patients… these are young people who cannot be easily addressed and for that we need someone that can help us (a psychologist) as well as to establish trust. Because some of them tell you the truth directly about what is going on, but others do not express it. But you can notice… sometimes when I look at a young man who is sad, isolated, unhappy…” (Female nurse)

### “Dealing with frustration and powerlessness”

When we explored feelings and emotions towards young help-seekers, the informants mentioned that their key reaction was one of frustration of not being able to help. Some reported that they felt incompetent, and they felt bad when they could not help. They perceived they had nothing to offer, even if they listened and tried to give advice.

“I feel like my hands are tied… I always have the feeling inside that I didn’t do anything.” (Female doctor)

The feeling of powerlessness was a reaction expressed in most of the interviews. Informants expressed feelings of sorrow and sadness when they could not help, and frustration at not being able to solve the patient’s problems, and therefore they felt that they were not living up to expectations.

Meetings patients who expressed a wish to die was difficult and distressing.

“It’s hard to listen to someone saying that he or she wants to die… it’s a shock when someone doesn’t want to live, and it’s worse when it’s a young person.” (Female doctor)

### “Barriers to helping young people”

Limited time, insufficient economic and human resources and lack of privacy in the consultation room were some factors described as barriers to helping young suicidal patients. The informants complained about receiving a great number of patients every day, they felt that they needed more training in mental health issues. They expressed a need to have a psychiatrist or psychologist in the PHC centre. Some of them reported that if they referred a patient to some other service elsewhere, the patient might not go there. Several nurses and doctors thought that patients who had already been engaged in a trustful relation with PHC staff would be reluctant to go to another health service. For that reason the nurse or the doctor would try to make a follow-up at home after having referred a patient.

“Here in the primary health care centre, time is our big problem… really we do not have enough time to address problems that teens may have.” (Female nurse)

Despite the fact that there exists a national guideline on how to manage patients with suicidal problems, most nurses and doctors were not familiar with the guide. Some of them who knew about but still did not use it.

Another barrier mentioned by nurses and doctors was the difficulty in communicating with young people. Difficulties in talking more openly about their problems were perceived as more common among men. Another barrier for nurses and doctors was difficulty in communicating with young people. It was mentioned that in reality it took two, three or more consultations to get to know a patient’s problem.

The lack of knowledge in how to manage these patients was highlighted as a key aspect. With better skills in detecting potential suicide patients, it would be possible to prevent attempts:

“Sometimes mental health is a matter of caring. Many patients come here complaining of pain, headache, but when I ask if that pain is not because of problems with her husband or her sons then the patients just laugh… so I know that I need to ask for other reasons behind that “pain” because there are patients that openly speak about their problems but there are also patients who do not…”(Male doctor)

An important aspect that doctors identified as a barrier was that according to law they are not allowed to be alone with a patient under 18 years of age. In those cases another adult must be present; it can be a relative or a nurse, according to the decision of the patient, but it influences the consultation and can make it harder for the patient to talk freely.

### “Identified needs for suicide prevention”

In the interviews the participants came to realize that young patients, prior to a suicide attempt, might visit the PHC presenting with symptoms other than suicidal problems, such as somatic complaints, for example headache or back pain. With more time for each patient, they were convinced that they would be able to look deeper into the reasons behind the somatic symptoms and explore aspects other than just physical symptoms. They also emphasized the need for more training in mental health and specifically in the management of suicidal patients. Some of them already had suggestions on what they could do, for example organising therapy groups. They emphasized training in skills on how to work with young people at risk of suicide, and that this training should include all staff.

“Because you [as a doctor] need to work more with the patient. As I say, “I’m no psychologist,” but I always look into how to investigate a little more, because there are always problems at home.” (Female doctor)

“I think I do my therapy because sometimes people complain that I spend too much time with every patient, but I think it’s good to talk with my patients… if I will be only prescribing medication, for me, that is not a quality consultation.” (Female doctor)

On the other hand, when they were able to help in one way or another, for example through listening, reaching through and calming down the patient, they felt relieved. One doctor commented on the vignette:

“We [should] listen and provide them with all we can, if they want to talk. It would be good to first start talking, give them advice and wait - not just let it go just like that… I would go to his family [and tell them] to look after him. Also I would call someone [a psychologist] to guide us. So you do everything that you can do, and then you feel relieved to be able to help… yes, maybe a little late, but better late than never, I think.” (Female doctor)

The doctors and nurses strongly asked for a stepped-care program so they would know how to manage patients at different risk levels.

They also suggested that adolescents needed a place to meet and share their problems, and to have regular meetings in the PHC centre would make young people feel that PHC is the right place to go when they have mental health problems. To arrange different activities together with the community, such as an adolescents’ club, or a small workshop, would be one way of making the PHC more attractive for young people.

“As a health centre we need to re-activate the teen club with the idea of encouraging activities for young people, sports, music, meetings between the clubs, so that will help them to do other things, like having their minds occupied also at school, giving them small workshops or inviting them to come for the different workshops and activities.” (Female doctor)

The informants considered that mental health problems among young people should be a responsibility shared with other agencies, not only a matter for the Ministry of Health. They asked for the active participation of other institutions and Non-Governmental Organizations (NGOs) to work together with them.

The participants reflected that the public health system should form a holistic whole and should in practice constitute an integrated system. This means seeing the patients from different perspectives in the different stages of life. Such a process was not considered possible without the participation of the community, who could help identify those with problems, and therefore a close collaboration with the PHC was felt to be important.

## Discussion

This study revealed a number of barriers in providing appropriate care to young people with mental health problems in PHC in Nicaragua. At the same time the interviewed PHC professionals suggested different ways to improve the treatment, and they acknowledged that they lacked sufficient knowledge and competence. Our informants agreed that they were not properly prepared to handle mental health problems, and they felt this as a challenge to their professional identity. Frustration made them at times ignore signs of mental health problems in their patients and sometimes even reject help-seeking young people.

Misconceptions among staff about suicidal behaviours have been reported in several studies [12,22-24]. Milton and co-authors showed that only one out of 52 general practitioners considered suicide to be preventable [13]. Our study also shows constricted thinking to be common. Some of our informants were also occupied by the contradiction between being trained to save lives and having to cope with young and seemingly healthy people who wish to die.

The barriers preventing health professional from being able to help were reflected in feelings of frustration, powerlessness and sadness. Similar findings have been described by, for example, Anderson and co-workers, who reported experiences of frustration to be important obstacles in dealing with young people with suicidal behaviour [11]. The informants were concerned about difficulties in openly discussing sensitive matters with their young patients, in accordance with the findings of Taylor and co-workers [6], who found that despite variations in health care systems and settings, communication between patients and staff was everywhere a key issue in improving services, user satisfaction and treatment adherence.

Overall, the health professionals in our study called for more training, similar to what has been reported by, among others, Slaven and Kesely [25]. Numerous studies have concluded that continuous training, adapted to different settings and resources, is a core aspect for health care professionals in order to perform better in the area of mental health and suicide prevention [26,27]. Huband and Tantam [8] specifically suggest training in counselling or psychotherapy, allowing staff to modify their attitudes by reducing defensive attribution and improving control of their own anxiety. Our informants also called for guidelines on how to manage patients with suicidal problems, and they even had suggestions for a stepped-care model to improve management of these patients. Strangely enough, none of the health care professionals in our study were aware of the existence of the national guidelines for the management of suicidal patients that was launched in 2003 [16]. Unfortunately, this gap between publishing programs and guidelines and having them implemented is a well-known problem worldwide.

Many informants held the view that reasons for young people attending PHC were confined to reproductive health issues. When the vignette was introduced in the interviews, they started to reflect more openly on other possible health problems among young people. Interestingly, our informants expressed few negative feelings towards young people with suicidal problems, in contrast to what has been reported in other studies (see for example: [9,11]. Instead, reluctance to deal with suicidal problems seemed more likely to stem from feelings of incompetence rather than from negative attitudes.

Taken together, feelings of incompetence and constricted thinking among the health care professionals made them prone to refer any young person with mental health problems to some other member of staff; this was evident with both among nurses and doctors. We used in our analysis the metaphor of a “hot potato” to illustrate this flow of internal referrals.

Studies have shown that suicide victims are likely to make contact with health care professionals shortly before they commit suicide. One study found that 87% of cases made contact with a health care professional within one month prior to suicide [28]. Another study has shown that such contacts increase in frequency throughout the months prior to committing suicide [4]. Despite their limitations, doctors and nurses acknowledged the benefits that a well-functioning PHC-system could have for patients with suicidal problems. They felt that they as PHC staff should be able to providing the “first help” for young people with suicidal thoughts must be a priority. They compared this situation with other medical emergency situations and felt that suicidal behaviour should be perceived as a similar emergency situation.

One limitation of this study was that we did not interview hospital staff about their perceptions of young people showing suicidal behaviour. However, there are reasons to believe that they share similar difficulties with those working at a PHC level. Evidence from a study conducted in United Kingdom showed that hospital staff also experienced frustration and mixed feelings in managing suicidal patients [11]. From our research findings theoretical generalizations can be drawn and they might be applicable in settings with similar characteristics as ours, such as LMIC, urban area and free services at PHC.

A review of mental health systems in low- and middle-income countries concerning management at PHC level argued that mental health services in primary health care should include diagnosis and treatment, but also strategies to prevent mental disorders and ensure that primary health care workers are able to apply psychosocial and behavioural skills, for example interviewing, counselling and interpersonal skills [2].

## Conclusion

The conclusion that we draw from the present study is that PHC staff in Nicaragua clearly acknowledged that providing mental health services to young people is important. Despite all constraints, the primary health care providers showed a willingness to improve their own skills in identifying and treating young people with suicidal behaviour. However, they felt that they lacked training and competence to handle mental health issues in general and specifically suicidal problems among young people. There is a need to design and implement policies to improve management of mental health problems as an integrated part of PHC services.

### Suggestions and implications for clinical practice

In Nicaragua reproductive health problems are probably the most common reason for attending PHC but this might provide an opportunity to reach young people’ and respond also to their mental health needs and to prevent suicidal behaviour.

Efforts must be made to establish continuous educational programs for primary health care staff on how to identify, manage and prevent mental health and suicidal problems among young clients.

The existing national guidelines for the management of suicidal patients must be made available and visible in a clear and appealing way to PHC staff. All this might help to avoid mismanagement of young suicidal patients such as the “hot potato” pattern of handing over responsibility to others where patients might be lost in the referral chain.

## Competing interests

We declare we have no competing interests.

## Authors’ contributions

CO designed the study, conducted all the interviews and was responsible for the initial coding, analysis and drafting of the manuscript. KD participated in the design, analysis and drafting of the manuscript. GK participated in the analysis and drafting of the manuscript. All the authors read and approved the final manuscript.

## Pre-publication history

The pre-publication history for this paper can be accessed here:

http://www.biomedcentral.com/1471-2296/15/129/prepub
